# *In silico* Prediction of Sex-Based Differences in Human Susceptibility to Cardiac Ventricular Tachyarrhythmias

**DOI:** 10.3389/fphys.2012.00360

**Published:** 2012-09-14

**Authors:** Pei-Chi Yang, Colleen E. Clancy

**Affiliations:** ^1^Department of Pharmacology, University of CaliforniaDavis, CA, USA

**Keywords:** arrhythmia, LQT, sex-differences, female, sex hormones

## Abstract

Sex-based differences in human susceptibility to cardiac ventricular tachyarrhythmias likely result from the emergent effects of multiple intersecting processes that fundamentally differ in male and female hearts. Included are measured differences in the genes encoding key cardiac ion channels and effects of sex steroid hormones to acutely modify electrical activity. At the genome-scale, human females have recently been shown to have lower expression of genes encoding key cardiac repolarizing potassium currents and connexin43, the primary ventricular gap-junction subunit. Human males and females also have distinct sex steroid hormones. Here, we developed mathematical models for male and female ventricular human heart cells by incorporating experimentally determined genomic differences and effects of sex steroid hormones into the O’Hara–Rudy model. These “male” and “female” model cells and tissues then were used to predict how various sex-based differences underlie arrhythmia risk. Genomic-based differences in ion channel expression were alone sufficient to determine longer female cardiac action potential durations (APD) in both epicardial and endocardial cells compared to males. Subsequent addition of sex steroid hormones exacerbated these differences, as testosterone further shortened APDs, while estrogen and progesterone application resulted in disparate effects on APDs. Our results indicate that incorporation of experimentally determined genomic differences from human hearts in conjunction with sex steroid hormones are consistent with clinically observed differences in QT interval, *T*-wave shape and morphology, and critically, in the higher vulnerability of adult human females to Torsades de Pointes type arrhythmias. The model suggests that female susceptibility to alternans stems from longer female action potentials, while reentrant arrhythmia derives largely from sex-based differences in conduction play an important role in arrhythmia vulnerability.

## Introduction

Female sex is a determinant of susceptibility to inherited and acquired long-QT syndrome and associated with Torsade de Pointes (TdP) arrhythmias (Pham and Rosen, 2002; Nakagawa et al., 2005; Furukawa and Kurokawa, 2007; James et al., 2007; Lowe et al., 2012). Studies have shown both genomic differences in ion channel expression in males versus females and sex-specific acute effects of sex steroid hormones that modulate ion channels (Bai et al., 2005; Nakagawa et al., 2005; Verkerk et al., 2005; Xiao et al., 2006; Furukawa and Kurokawa, 2007; Sims et al., 2008; Yang et al., 2012). Recent clinical and experimental studies suggest that differences in vulnerability to some arrhythmias may arise directly from these fundamental sex-based differences in cardiac tissue (Di Diego et al., 2002; Fish and Antzelevitch, 2003; Gaborit et al., 2010; Nattel et al., 2010). However, teasing out the key determinants of sex-based differences in arrhythmia vulnerability is an exceedingly complex problem since it is difficult to reveal how specific components (i.e., acute effects of hormones versus genomic effects underlying variable ion channel expression) contribute to differences in electrophysiological substrate and vulnerability to arrhythmia.

Recent work from the Demolombe lab has provided the first comprehensive glimpse into sex-based differences in cardiac ion channel composition and distribution in non-diseased human hearts (Gaborit et al., 2010; Nattel et al., 2010). A high-throughput quantitative approach revealed genome-scale differences in expression of key genes encoding cardiac ion channels and transporters subunits in human epicardial and endocardial male and female tissues. Of note were sex-based differences in expression of genes encoding channels that determine conduction and repolarization. Female hearts had reduced expression of the K(+)-channel subunits HERG, minK, Kir2.3, Kv1.4, KChIP2, SUR2, and Kir6.2, and lower expression of connexin43 and phospholamban compared to males. Experiments demonstrated concurrence of changes between gene and protein-expression for HERG, minK, Kv1.4, KChIP2, IRX5, Nav1.5, and connexin43. The findings reported in this study suggest a potential vulnerability of females to repolarization abnormalities, but did not show the functional effects of genomic-based differences to determine is they were sufficient for observed clinical differences in males and females.

Although females are at increased risk for inherited and acquired long-QT syndrome and TdP compared to males, risk and QT interval fluctuations have been shown to change with phases of the menstrual cycle in some studies, suggesting a role for fluctuating hormones (Furukawa and Kurokawa, 2007; James et al., 2007). During the menstrual follicular phase (prior to ovulation), QT interval is longer than in the luteal phase (following ovulation) when progesterone is increased (Nakamura et al., 2007). Moreover, susceptibility to drug-induced arrhythmias is exaggerated in the late follicular phase where estrogen is highest (James et al., 2007). Arrhythmic events associated with acquired and inherited LQTs are reduced during phases where progesterone is high (Janse de Jonge et al., 2001; Nakagawa et al., 2006).

Recent studies have shown that progesterone and estrogen affect cardiac electrophysiology in an acute manner via effects on ion channels (James et al., 2007). Progesterone results in enhancement of the slow delayed rectifier K^+^ current (*I*_Ks_) and rapid shortening of Action potential duration (APD; Nakamura et al., 2007). The effects of progesterone are mediated by nitric oxide (NO) released via non-genomic activation of endothelial NO synthase (eNOS; Furukawa and Kurokawa, 2007). Estrogen, on the other hand, primarily affects the rapidly activating component of the delayed rectifier K^+^ current, *I*_Kr_ via direct binding to the channel and reducing current (Kakusaka et al., 2009).

In males, testosterone increases during puberty, a phase during which a reduction in QT intervals and arrhythmia susceptibility is observed compared to females (Bidoggia et al., 2000). The QT interval then gradually increases with age, apparently corresponding to age related reduction in serum testosterone (Bidoggia et al., 2000). Men are much less likely to develop TdP arrhythmias in the presence of congenital or drug-induced prolongation of QT interval (Pham et al., 2002; El-Eraky and Thomas, 2003; James et al., 2007). Experiments in female animals have demonstrated that administration of testosterone reduces pro-arrhythmia induced by dofetilide (Pham et al., 2002). The action of testosterone, like progesterone, is mediated via eNos production of NO and modification of channel kinetics (Bai et al., 2005).

In order to begin to understand the contributions of various components that contribute to sex-based differences in arrhythmia risk, we utilized experimental data describing the differences in male and female expression of ion channel gene transcripts to build male and female human cardiac cell models via modification of the O’Hara–Rudy human ventricular cell model. Acute effects of physiologically relevant human concentrations of sex steroid hormones testosterone, progesterone, and estrogen were then applied. The male model incorporated measured effects of two concentrations of testosterone (adolescence and senescence) on the critical plateau currents *I*_Ks_ and L-type Ca^2+^ current (*I*_CaL_). The female model included effects of physiological concentrations (at different points in the menstrual cycle) of progesterone on *I*_Ks_ and estrogen on *I*_Kr_. Simulations revealed the conditions (i.e., pacing, drugs) required for sustained reentrant arrhythmias. The model simulations allowed us to explore the complex non-linear interplay between processes on disparate time scales (acute and genomic effects of hormones) that contribute to sex-based arrhythmias.

To realize the most basic biological mechanisms underlying sex-based differences in male and female hearts and their influence on arrhythmia proclivity is the first necessary step that must be taken to ultimately lead to development of specific diagnosis criteria and distinct therapeutic targeting of cardiac disease in males and females. Basic understanding of mechanisms is required to envision a future when women will have designated sex-based parameter regimes and not be classified as “atypical” because they fall outside of the “normal” range designated for male physiology and pathology.

## Materials and Methods

### Cellular simulations

The model formulation for the baseline male endocardial cell was the O’Hara–Rudy endocardial model cell (O’Hara et al., 2011). Experimentally measured differences in mRNA expression were expressed relative to male endocardial cells (male endocardial is always “1”). The computational models for male epicardial, female epicardial, and female endocardial cells were derived by altering channel conductances based on measurements in these cell types relative to the published O’Hara–Rudy endocardial model cell. Unless otherwise indicated, simulated action potentials (APs) were recorded at the 1000th paced beat (BCL = 1000 ms) in single endocardial and epicardial cells using the index (calculated from the average experimentally obtained value) as indicated in Table S1 in Supplementary Material. The numerical method used for updating the voltage was forward Euler. Source code is available upon request. Full detailed Methods accompany this paper in the Supplementary Material.

## Results

### Effects of sex-based differences in human ion channel expression on action potentials

**Figure 1:**
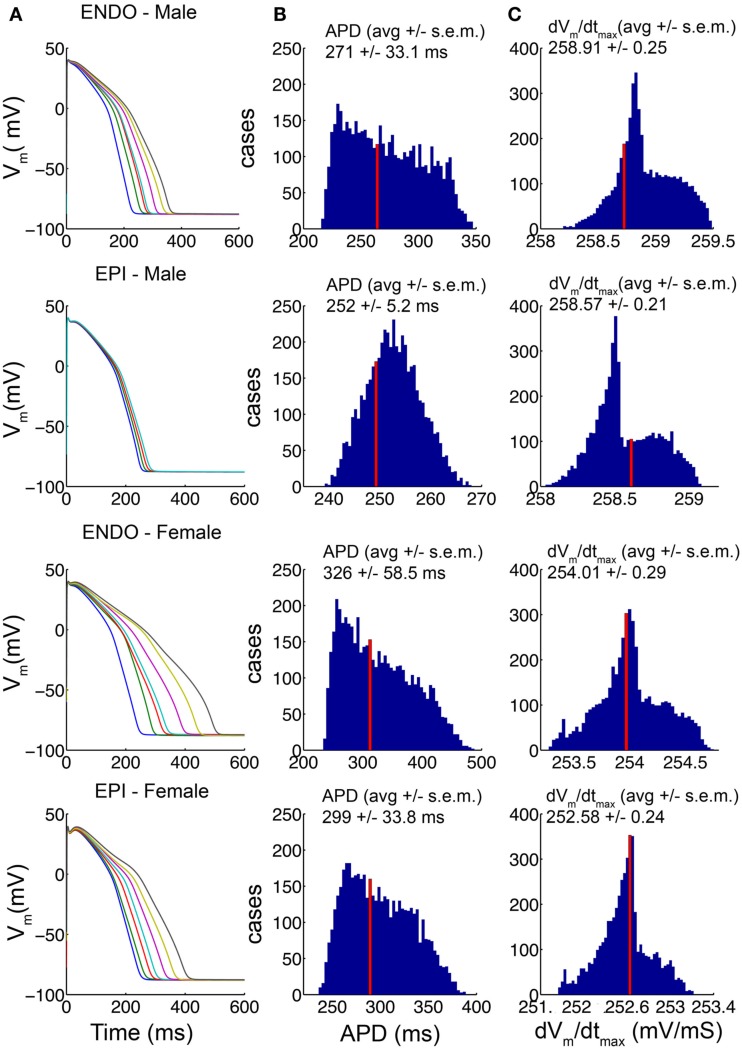
**Channel conductances chosen randomly from within the standard deviation of experimental data (Table S1 the Methods in Supplementary Material) for 5000 simulations of human ventricular cell**. The 1000th paced beat at a cycle length of 1000 ms is shown in endocardial and epicardial cell types. Action potentials (APs) from male and female models are shown **(A)**. The distributions of APD variations **(B)** and the distributions of maximum upstroke velocity **(C)** for each model are shown. The average values are indicated. The index values derived from the mean experimental values are indicated by the red bars.

In a recent study from the Demolombe group (Gaborit et al., 2010), a high-throughput quantitative approach was used to determine the expression level of key cardiac ion channel genes in epicardial and endocardial ventricular tissue from non-diseased explanted male and female human hearts. This study revealed critical differences in male and female expression of ion channel gene transcripts that were validated by and consistent with resultant protein-expression for all of the subunits that were examined. We utilized these data (shown in Table S1 in the detailed Methods in the Supplementary Material) in construction of “male” and “female” human heart cell computational models by scaling the conductances of the corresponding currents in the O’Hara–Rudy human ventricular cell model as described in the accompanying detailed Methods in the Supplementary Material. The baseline O’Hara–Rudy model formulation was assumed to be the “average male” endocardial cell, while male epicardial and female endocardial and epicardial conductances were scaled relative to this baseline. Because much variability exists within the experimental data for each channel transcript, we constructed 5000 distinct models with combinations of ionic conductances based on a random value chosen from within the standard deviation of the experimental data. Regardless of the parameter sets that were used in the models, a clear prediction emerged from the model simulations as shown in Figure 1: consistent with the observation that females have longer QT intervals than males, “female” cell reconstructions had longer APD than “male” cell reconstructions for both endocardial and epicardial cell types. In column A of Figure 1, the 1000th paced beat at a cycle length of 1000 ms is shown for seven combinations of ion channel conductance parameters picked at random within the standard deviation of the data in male and female endocardial and epicardial cell types as indicated. APDs and maximum upstroke velocities (dvm/dtmax) compiled from 5000 distinct cases (again with combinations of ion channel conductance parameters picked at random from within the standard deviation of the data) in male and female models are shown columns B and C, respectively. The “index” values indicated by the red vertical lines, were generated by using the mean experimental data values for each component in scaling the ion channel conductances in the model. That the cellular level parameters, APD and upstroke velocity, derived from the average ionic conductance parameter values were within the middle of the distribution for all cell types indicates that these are reasonable representations of an “average” cell in each class. This analysis also serves as a test of sensitivity of the model behaviors to the underlying parameters, which indicated clear sex- and cell-type-based trends for all parameter combinations, providing confidence in the model predictions. The “index” models for male and female endocardial and epicardial cells were used for the remainder of simulations in this study.

### Simulated genomic-based differences in combination with hormone effects on action potentials in single cells

We next utilized the “index” APs (indicated by red lines in Figure 1) and added additional acute effects of sex steroid hormones in the model to predict the combined effects of genomic-based differences in expression of key cardiac channels with human physiological concentrations of sex hormones on human APD. In recent studies, progesterone enhanced *I*_Ks_ (Nakamura et al., 2007), while testosterone primarily increased *I*_Ks_ and inhibited *I*_Ca,L_ (Bai et al., 2005). Application of estradiol significantly suppressed *I*_Kr_ current (Kurokawa et al., 2008). Based on the experimentally observed effects of physiological concentrations of sex steroid hormones on these channels, we modified the kinetics and conductance of model ion currents (described in Methods of Supplementary Material).

**Figure 2:**
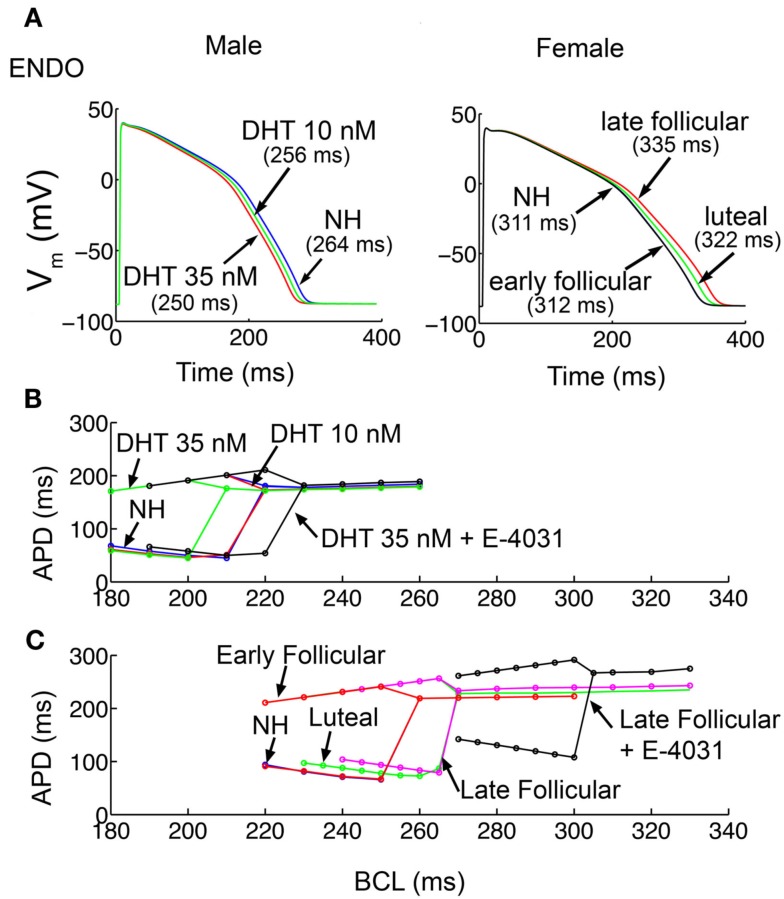
**Simulated action potentials duration (APD) and APD alternans**. **(A)** APD for the 1000th paced beat at a cycle length of 1000 ms in single endocardial cells. APDs with no hormone (NH; Table S1 the Methods in Supplementary Material) and two physiological concentrations of DHT (10 and 35 nM) in male cells are shown in left. Estradiol and progesterone at different physiological concentrations corresponding to three stages of the menstrual cycle were added in female cells (right). **(B)** Male epicardial cells with DHT treatment developed alternans at fast pacing rates, between cycle lengths 180 and 210 ms. **(C)** In female epicardial cells alternans developed at a much slower basic cycle length during the late follicular phase (pink) compared to luteal (green) and early follicular (red). *I*_Kr_ block increased susceptibility to alternans development in male (black in **B**) and dramatically in female models (black in **C**).

Upon acute application of two physiological concentrations of testosterone (dihydrotestosterone DHT) 10 and 35 nM shown in Figure 2A (left), reflecting normal low and high ranges in post-pubescent pre-senescent males (Dorgan et al., 2002), the model simulations predict APD shortening in endocardial cells compared to no hormone (NH, 264 ms) by 3.0% (256 ms) and 5.3% (250 ms) at 10 and 35 nM testosterone, respectively. The model predictions also suggest modest APD shortening in epicardial cells (Figure S1A in Supplementary Material) with testosterone compared to NH (249 ms) by 2.8% (242 ms) and 4.0% (239 ms) at 10 and 35 nM testosterone, respectively.

Figure 2A (right panel) shows the combined effects of female channel gene expression and the hormones 17β-estradiol (E2) and progesterone during the early follicular, late follicular, and luteal phases of the menstrual cycle. During the early follicular stage, E2 = 0.1 nM, progesterone = 2.5 nM, during the late follicular stage, E2 = 1.0 nM, progesterone = 2.5 nM, and during the luteal phase, E2 = 0.7 nM, progesterone = 40.6 nM (Janse de Jonge et al., 2001). As see in Figure 2A right panel, the simulations predict the longest APD in the late follicular phase in endocardial cells (335 ms), considerably longer than in the early follicular (312 ms – 6.9% reduction) or luteal phase (322 ms – 3.9% reduction). This trend was consistent for model epicardial cells (Figure S1B in Supplementary Material), where late follicular phase (313 ms), was longer than early (290 ms – 7.4% reduction) and luteal phase (301 ms – 3.8% reduction).

Due to the predicted sex-based differences in APD, we postulated that APD restitution, a quantity describing adaptation of APD to the preceding diastolic interval (DI), and the propensity of the cell to develop arrhythmogenic rhythms such as alternans (alternating long-short pattern of APD) would be different between the sexes. To examine the sex-based rate-dependence of cellular dynamics, cells were paced at constant frequency as indicated for 1000 beats (see Methods in Supplementary Material) for male and female cells with hormone combinations as shown. As shown in Figure 2B, the virtual male cell with 10 nM testosterone (red) has indistinguishable dynamics compared to NH (blue), but required substantially faster pacing to develop alternans in the presence of 35 nM (green) testosterone [faster than 292 beats per minute (BPM)], indicating that testosterone is protective. When the *I*_Kr_ blocker E-4031 (10 nM) was administered (black) to simulate the common situation of promiscuous hERG block, there was a right shift of the restitution curve, indicating the development of alternans at slower, more physiologically attainable, albeit very high, frequencies (onset of alternans at 267 BPM).

In female model cells (Figure 2C), alternans developed at considerably slower frequencies, with similar onset for NH compared to the early follicular phase, with a marked right shift of the curve observed for the luteal phase (green) and the late follicular phase (pink). *I*_Kr_ block (E-4031 10 nM) dramatically increased susceptibility to alternans (at 197 BPM) in the female simulations as indicated by the right shift of the black curve. These results indicate that the female cells in the presence of *I*_Kr_ block, particularly in the late follicular phase of the menstrual cycle when estradiol is highest, are considerably more vulnerable to the arrhythmogenic alternans rhythm. Restitution slopes are shown in Figure S2 in Supplementary Material.

### Simulation of tissue-level effects of genomic differences and hormones

**Figure 3:**
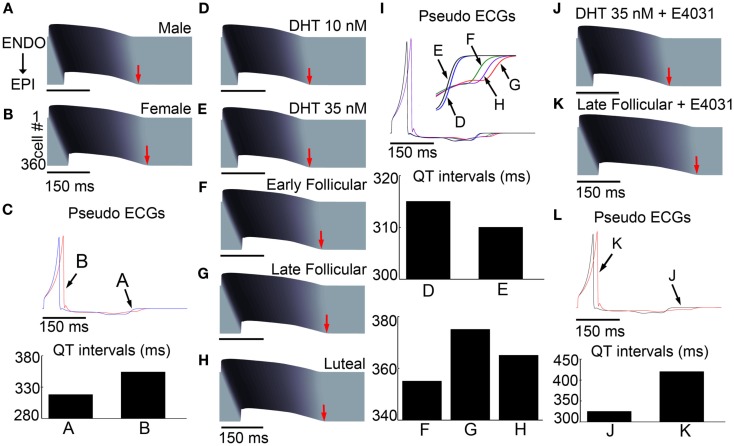
**Sex-based differences in human cardiac ion channels altered myocyte electrophysiology in transmural 1-dimensional tissue models**. Simulated action potentials (APs) at 1000 ms pacing frequency in a 3.6 cm cardiac fiber. Time is shown on the *x*-axis and voltage on the *z*-axis. The 500th paced beats are shown. **(A)** Simulated male fibers with no hormone. **(B)** Female fibers with no hormone. **(C)** Pseudo ECGs show the genomic effects on QT intervals for male [**(A)** – blue] and female [**(B)** – red] models. **(D–H)** Simulated combined effects of estradiol and progesterone during the menstrual cycle and DHT on APs. **(I)** The virtual ECGs (the 500th beat) show QT intervals change during the menstrual cycle and two physiological concentrations of DHT, indicated by black arrows. **(J,K)** Disparate responses to *I*_Kr_ channel block in the late follicular phase versus with DHT. **(L)** The computed ECG (the 500th beat) shows that QT interval is substantially longer in the late follicular phase [**(K)** – red) than with DHT [**(J)** – black]. Ninety five percentage repolarization of last cells indicated red arrows.

We next computed the effects of sex-based genomic differences alone in a one-dimensional transmural strand of coupled cells comprising epicardial to endocardial regions (detailed explanation of construction of the tissue is in the Methods in Supplementary Material) to determine the sex-based genomic effects in an electronically coupled male (A) and female tissue (B; Figure 3). We also computed spatial gradients of depolarization and repolarization to generate an electrogram to allow us to compare “ECG” parameters between male (blue) and female (red) virtual tissues (Figure 3C).

Action potentials were initiated via a stimulus applied to the first endocardial cell that propagated to the last epicardial cell along the 360 cell tissue. The *T*-wave in Figure 3C for both male and females is inverted and poorly pronounced indicating that the endocardial cells fired first and repolarized first, even though endocardial cells have a longer intrinsic APD in isolation. The flat *T*-wave indicates that electronic coupling in the tissue is sufficiently strong to damp large repolarization differences between epicardial and endocardial cells so that there is very little dispersion of repolarization. Importantly, the simulation also shows a prolongation of repolarization in the female compared to male, consistent with the longstanding observations that females have longer QT intervals than males (Jose and Collison, 1970; Huikuri et al., 1996; Burke et al., 1997; Stramba-Badiale et al., 1997; Bidoggia et al., 2000; Smetana et al., 2002). Sex-based differences in conduction velocity are shown in Figures S3 and S4 in Supplementary Material.

In Figures 3D,E, we used the transmural tissue model to simulate effects of low and high physiological concentration of testosterone on APD in simulated transmural one-dimensional tissue. The model predicts that testosterone-induced faster repolarization and consequent QT interval reduction (3D) to 315 ms (0.94% shortening – 10 nM DHT) and 310 ms (2.5% shortening – 35 nM DHT) compared to NH (318 ms) in male tissue in Figure 3A.

Clinical studies have suggested that the QT intervals fluctuate during the menstrual cycle, suggesting that progesterone may reverse effects of the estradiol-induced QT prolongation (Nakagawa et al., 2006). Figures 3F–I represents the results of simulations in a 1D cable at combined hormone concentrations observed during various phases of the menstrual cycle. Simulations show a QT interval that is longest (375 ms) in the late follicular phase (3G) compared to 365 ms in the luteal phase and 355 ms in the early follicular phases, in agreement with the clinically observed QT shortening (≈10 ms shortening in the luteal phase compared to the follicular phase; Nakagawa et al., 2006). The models demonstrate that despite the presence of estradiol (0.7 nM) during the luteal phase, high progesterone (40.6 nM) results in luteal phase shortening of APD and a QT interval (on the pseudo ECG) reduction of 2.7% (from the late follicular phase).

### Combined effects of hormones and *I*_Kr_ channel blocking drugs

Clinical and experimental studies have shown that females are especially susceptible to QT interval prolongation by *I*_Kr_ blocking drugs (Kurokawa et al., 2008). This finding may partially explain why females are more prone to drug-induced arrhythmias (Makkar et al., 1993; Lehmann et al., 1996). Hence, we next tested the combined effects of hormone and the *I*_Kr_ channel blocker E-4031 on drug-induced QT interval prolongation. Experiments (Kurokawa et al., 2008) have previously shown that physiologically relevant application of estradiol increased hERG block by E-4031, whereas testosterone did not. Figures 3J–L illustrates a simulation in one-dimensional transmural tissue of coupled cells (360 cells) in the presence of 35 nM testosterone during E-4031 treatment compared to combined female hormones during the late follicular phase where estradiol is highest. With 10 nM E-4031, the simulated tissue-level pseudo ECG QT (panel L) is markedly shorter with testosterone application (black line; 325 ms) compared to the QT interval in the presence of female hormones (420 ms). These results are consistent with clinical observations that females experience more QT interval prolongation than males in the presence of *I*_Kr_ blocking drugs (Benton et al., 2000; El-Eraky and Thomas, 2003; Kurokawa et al., 2008).

**Figure 4:**
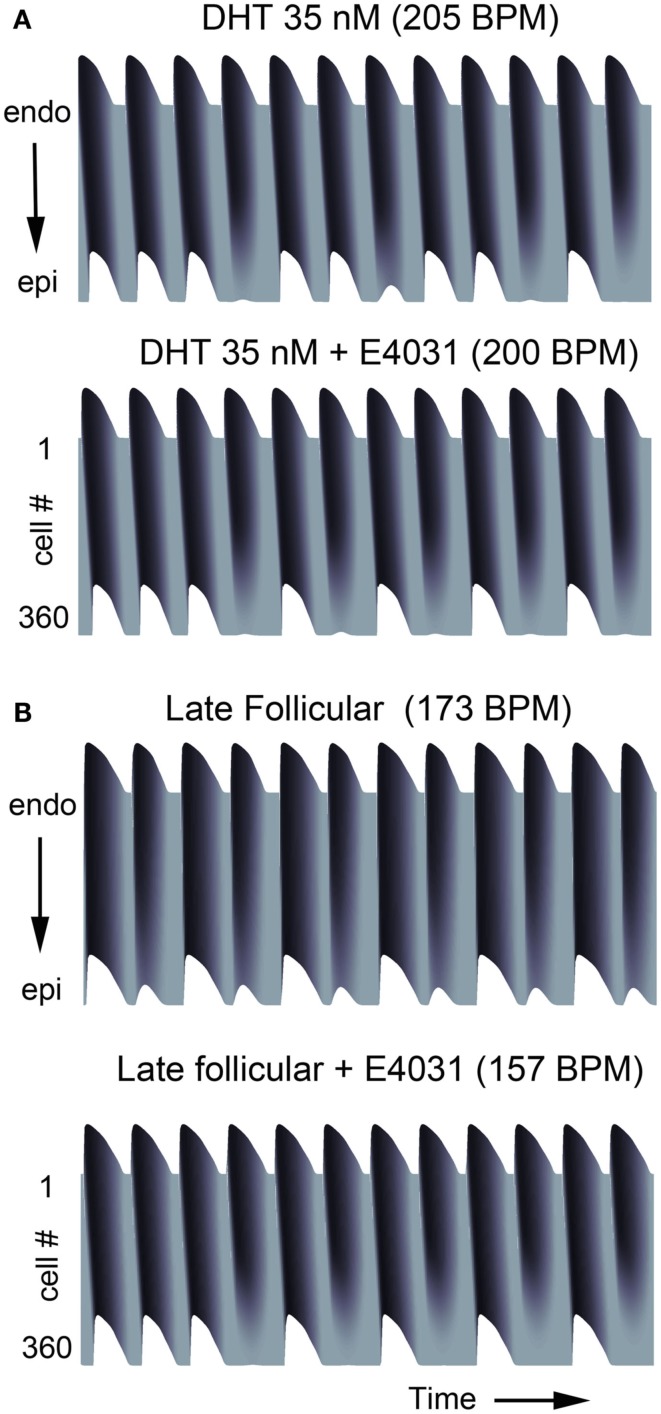
**Alternans occurs at slow pacing rates in female tissue**. Transmural tissue composed of epi-/endocardial ventricular cells were paced at various cycle lengths. **(A)** Male with DHT 35 nM and/or E-4031 10 nM. **(B)** Female in late follicular phase (top) and with E-4031 10 nM (bottom).

In Figure 4, we show that the model predicts the development of complex rhythms including alternans, “Wenckebach-like” rhythms and conduction block in transmural tissue composed of a one-dimensional strand of coupled cells during rapid pacing (see Materials and Methods for pacing protocol). Tissues composed of male cells required fast pacing frequencies to develop these rhythms in the presence of testosterone (Figure 4A, upper panel) and even simulated addition of the *I*_Kr_ blocker E-4031 did not overwhelm testosterone’s protective effect (Figure 4A, lower panel). Male tissues develop (Figure 4A) alternans around 205 and 200 BPM with drug addition. Consistent with the simulations in single cells showing steeper APD restitution in females (Figure S2 in Supplementary Material), female transmural tissue developed conduction block at considerably slower pacing rate compared to the male case. Female tissue during the late follicular phase generated alternans and Wenckebach phenomena at physiologically relevant frequencies of 173 BPM and 157 BPM with *I*_Kr_ blocker, where conduction block was also observed.

### Effects of sex-based genomic differences, hormones and *I*_Kr_ block on propensity to reentrant excitation in tissues

Critically timed stimuli can initiate self-sustaining spiral waves (Mines, 1914; Allessie et al., 1973) capable of degeneration into fibrillatory rhythms leading to sudden cardiac death. Thus, we sought to systematically determine the likelihood of arrhythmia induced by irregular spontaneous stimuli in tissue models of the human male and female heterogeneous ventricular myocardium. We tested vulnerability to persistent reentry induced by ectopic stimuli with physiologically relevant concentrations of sex steroid hormones in the presence and absence of the common clinical situation of *I*_Kr_ block by simulating a heterogeneous cardiac tissue containing an endocardial region (tissue columns 1–160) and an epicardial region (tissue columns 161–360) based on recordings from human tissue (Glukhov et al., 2010; Lou et al., 2011) as described in detail in the Methods in Supplementary Material. We also incorporated anisotropic effects by setting *D*_x_ and *D*_y_ such that the ratio of conduction velocity is 1:2 (Frank and Kranias, 2000). APD dispersion maps are shown in Figures S5 (male) and Figure S6 (female) in Supplementary Material. Following pacing for 50 beats (s1) at BCL = 1000 ms along the entire endocardial length, a premature stimulus (S2) was then applied in a 2.7 cm × 1.5 cm area on the top edge of the endocardial region within the vulnerable window (described in Methods in Supplementary Material).

**Figure 5:**
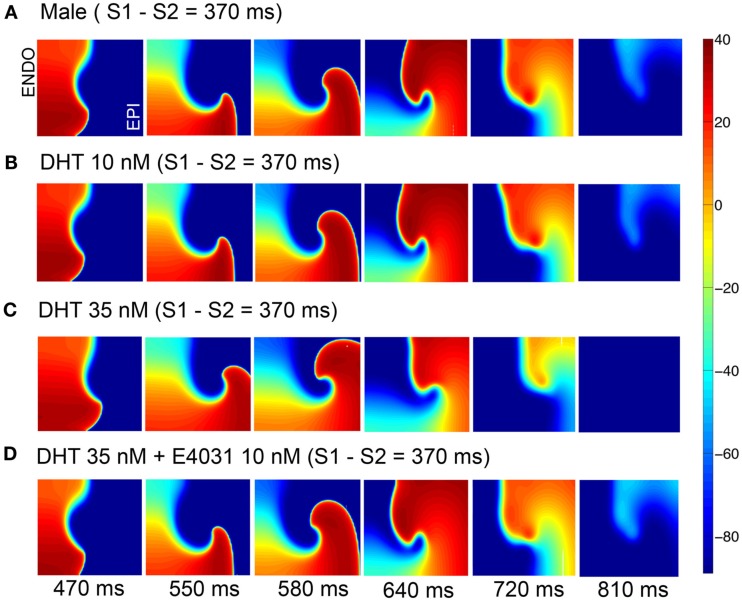
**Attempted induction of reentry in male heterogeneous tissue without (A) or with (B,C) DHT 10 or 35 nM in the presence of 10 nM E-4031 (D)**. Tissues (5.4 cm × 6.6 cm) were stimulated along left edge (endocardium) and propagated across the tissue followed by a premature stimulus applied in the endocardial region (see Materials and Methods). Six snapshots following application of DHT and/or drug at indicated time points. Voltages are indicated by the color gradient.

Figure 5 shows voltage snapshots in time as indicated following the application of the simulated ectopic beat via application of the premature stimulus S2 for four male cases. In the absence of hormones or drugs (A), an initiated wave propagates in a reentrant manner and makes only one turn (first row). In B, the same behavior is shown following testosterone (10 nM) application alone. With application of 35 nM DHT, the reentrant wave fails to make a complete turn (C), and application of the *I*_Kr_ blocker E-4031 (D), does not substantially worsen the situation – only one turn of the reentrant wave is observed.

**Figure 6:**
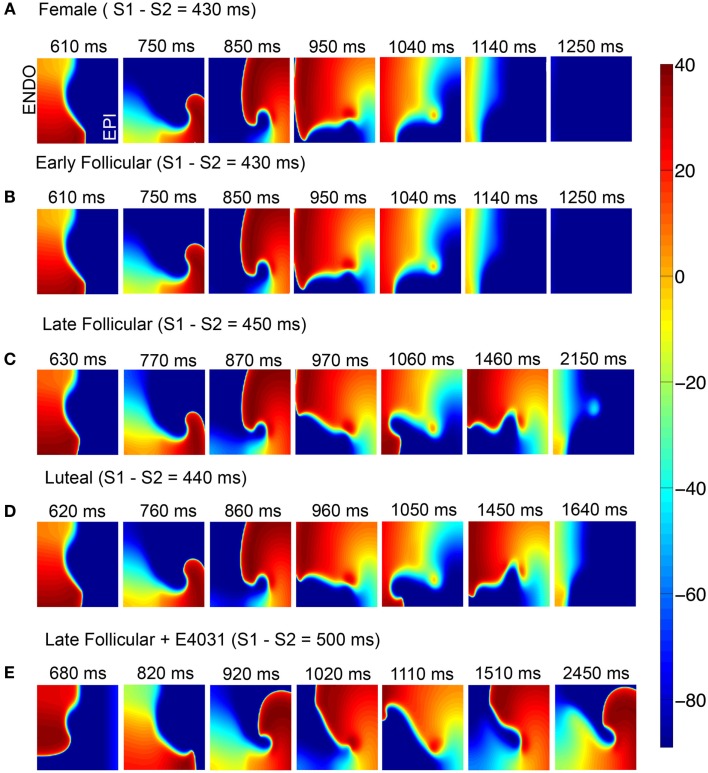
**Attempted induction of reentry in female heterogeneous tissues in the absence (A) or presence of female hormones (B–D) and *I*_Kr_ block during late follicular phase (E)**. Seven snapshots following application of hormones and/or drug at indicated time points. The protocol was as in Figure 5.

The outcome was starkly different in the female (Figure 6). In Figure 6A (top), the simulations suggest that in the absence of female sex steroid hormones, a single reentrant wave occurs and then extinguishes during the second reentrant cycle. A nearly identical pattern was observed during the early follicular phases (B). However, the late follicular phase (C) and the luteal phase (D) are apparently more vulnerable to reentrant activity – in these cases, the self-sustaining oscillations persist for more than 2 and 1.6 s, respectively. When E-4031 was applied during the late follicular phase (E), a premature stimulus resulted in the robust induction and persistence of a spiral wave. Sustained reentry was introduced in this condition that persisted indefinitely, suggesting female vulnerability to reentry is especially marked during the late follicular phase of the menstrual cycle. The early follicular and luteal phase with E-4031 results are shown in Figure S7 in Supplementary Material.

**Figure 7:**
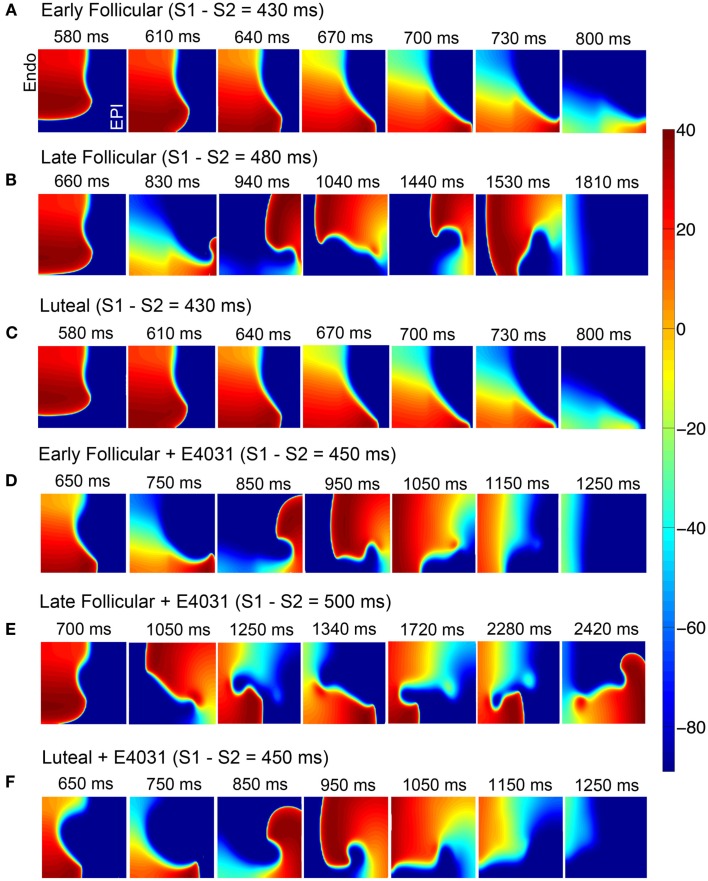
**During SNS stimulations simulated reentry wave on female heterogeneous tissues in the presence of female hormones (A–C) and *I*_Kr_ blocker (D–F)**. Time pointes and menstrual cycle phases are indicated. The protocol was as in Figure 5.

Because the experimental data (Nakamura et al., 2007) also showed that progesterone inhibited *I*_Ca,L_ with cAMP treatment, we next investigated how the effects of progesterone on *I*_Ca,L_ influence the reentrant behaviors. Figures 7A–C show that the reentry wave persists for about 1.81 s in the late follicular phases, was longer than in the early follicular and luteal phases (0.8 s). Figure 7E suggests that during late follicular phase with E-4031 application, a persistence of spiral wave was observed. However, the reentrant waves terminated around 1.25 s in both early follicular (Figure 7D) and luteal phases with *I*_Kr_ blocker (Figure 7F), suggesting protective effects of progesterone during cAMP-stimulations. The model also predicts the under SNS stimulation, progesterone-inducted APD reductions in single cell during the luteal phase shown in Figure S8 in Supplementary Material.

**Figure 8:**
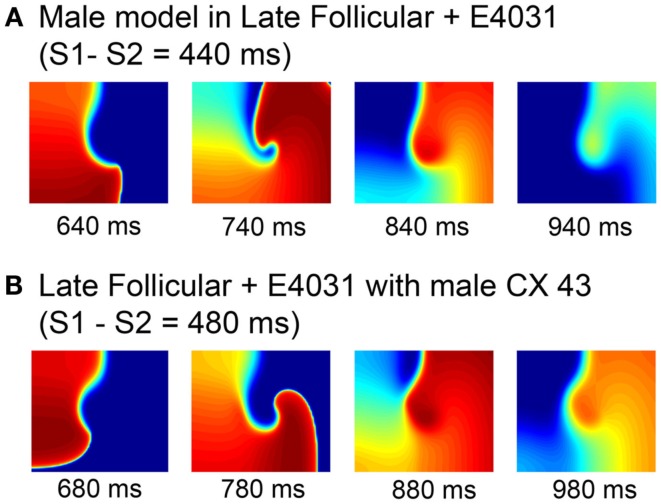
**Attempted induction of reentry in male heterogeneous tissues with female hormones and *I*_Kr_ block during the late follicular phase (A) and female heterogeneous tissues in the late follicular phase and E-4031 10 nM with measured male connexin 43 level (B)**. Four snapshots following application of hormones and/or drug at indicated time points. The protocol was as in Figure 5.

In order to isolate the mechanism of female susceptibility to reentrant arrhythmia, we made some targeted swaps of model components. As seen in Figure 8A, we first added female hormones (1 nM E2 and 2.5 nM progesterone) and the *I*_Kr_ blocker E-4031 to the “genetic” male model and tested for vulnerability to reentry using the S1–S2 protocol already described. The addition of drug and female hormones had the expected effect to increase the APD. But the faster male conduction velocity resulted in a rapid collision of the wave front with the wave tail after a single rotation. Similar behavior was observed when the genetic female model with female hormones corresponding to the late follicular phase was combined with the *I*_Kr_ blocker E-4031, but male connexin 43 level was the only swapped component (Figure 8B). Again, this simulation suggests that sex-based differences in conduction play an important role in arrhythmia vulnerability.

## Discussion

The first observation of sex-based electrophysiological differences on the ECG came from Bazett approximately 85 years ago (Bazett, 1920). Bazett (1920) noted that women had significantly longer QT intervals than men, despite having higher resting heart rates. Many studies have since confirmed Bazett’s observations of sex-based differences in the ECG (Jose and Collison, 1970; Rautaharju et al., 1992; Huikuri et al., 1996; Burke et al., 1997; Stramba-Badiale et al., 1997; Bidoggia et al., 2000; Smetana et al., 2002). It is thus surprising that even the most basic scientific investigations to reveal how sex-based biological differences influence cardiovascular-linked disease outcomes are just beginning to be undertaken. For example, investigations into how male and female hearts differ fundamentally in terms of gene expression and how sex steroid hormones modify heart function and cardiovascular risk are still in early stages. It is critical that such studies be undertaken and expanded now. It will be essential to effectively disseminate information and manage risk, diagnosis, treatment and follow-up for males and females with cardiovascular rhythm disorders. But, the sex-based mechanisms that underlie disease disparity in women and men must be determined before these effective prevention and treatment strategies can be effectively deployed.

Several studies have suggested that sex hormones affect cardiac repolarization by regulating gene expression and causing functional physiological changes in the heart, as well as through rapid acute effects on cardiac ion channels. In this study, we sought to use a computationally based approach to explore the complex non-linear interplay between genomic differences and acute fluctuating effects of hormones that contribute to sex-based differences in arrhythmias.

We validated our model predictions against surrogate markers of arrhythmia risk (e.g., prolongation of the QT interval in females versus males). Clinical studies have shown that females have longer corrected QT interval than males (420 versus 400 ms; Stramba-Badiale et al., 1997; Ebert et al., 1998; Nakagawa et al., 2005). Our simulations suggest that genetic differences in expression of key cardiac ion channels can explain sex-based differences in APD. Addition of sex steroid hormones exacerbated these differences, especially when comparing high physiologically realistic concentrations of the male hormone testosterone (35 nM) to the late follicular phase during the menstrual cycle when estradiol is highest and progesterone is low. Our model simulations predicted that increased APD in the female made the development of alternans and other arrhythmia-linked complex rhythms, including Wenckebach periodicity and conduction block, arise at slower, physiologically achievable heart rates, which may put females at particular risk.

We also sought to determine if sex-based differences in expression of key cardiac ion channel proteins and physiologically relevant concentrations of sex steroid hormones would *exacerbate or protect* against persistent self-sustaining reentrant arrhythmias, a clinically significant precedent event to lethal arrhythmias that have been observed with significantly higher incidence in women in the setting of acquired long-QT (Regitz-Zagrosek, 2006). We found that following application of a premature stimulus in the presence of female hormones during the late follicular phase with the *I*_Kr_ blocker E-4031 induced sustained reentry in the transmural tissue model. In addition, connexin43 play a key factor for sustained reentry. Connexin43 was reduced in females compared to males (Gaborit et al., 2010). Our study suggests that this sex-based difference is a key factor in female vulnerability of reentrant arrhythmia.

We previously undertook a study of sex-based differences in electrical properties of cells in response to acute hormone application in a guinea pig cardiac cell model. In the guinea pig computational cells, simulations predicted the protective effects of progesterone against drug-induced arrhythmias. However, contrary to the results of simulations in the guinea pig model, the human model predictions suggest that progesterone alone is not sufficient to reduce risk of TdP arrhythmias in females. This species disparity is explained by variability in K^+^ current expression, especially *I*_Ks,_ which is notably less prominent in humans (Virag et al., 2001; Grandi et al., 2010; O’Hara et al., 2011). The human model simulations suggest that progesterone is protective against estradiol-induced LQT syndrome only when concurrent sympathetic stimulation is present. This is because progesterone mitigates the increase in *I*_Ca,L_ that occurs via PKA phosphorylation subsequent to adrenergic activation.

### Limitations

It is important to note that β-adrenergic stimulation has also been shown to modify testosterone effects on *I*_Ca,L_ current. A limitation of this study is that we were not able to directly simulate these effects because the experimental data under physiological testosterone concentrations were not available. Even so, effects of sympathetic stimulation are expected to add to the protective effects of testosterone by further reducing *I*_Ca,L_ current in response to β-adrenergic activation leading to further reduction in APD and reduced potential for sustained reentrant activity. We were also unable to independently determine properties of a baseline male cell, and so began this study with the assumption that the O’Hara–Rudy model represents a baseline male model. The original model was based on majority male data (56%), but a substantial fraction was female (44%). We acknowledge this assumption as a limitation of the study, which may have led to an underestimate of male and female differences in electrophysiology and arrhythmia vulnerability.

Another limitation of this study is that we used mRNA expression data as a surrogate for actual electrophysiological recordings of ionic currents because such recordings are not yet available. Although most of the key changes to electrophysiological components included in our models were the subject of experiments that indeed demonstrated concurrence of changes between gene and protein-expression for HERG, minK, Kv1.4, KChIP2, IRX5, Nav1.5, and connexin43, we acknowledge that these data are less definitive than electrophysiological recordings.

## Conflict of Interest Statement

The authors declare that the research was conducted in the absence of any commercial or financial relationships that could be construed as a potential conflict of interest.

## Supplementary Material

The Supplementary Material for this article can be found online at http://www.frontiersin.org/Computational_Physiology_and_Medicine/10.3389/fphys.2012.00360/abstract

Supplementary Table S1 **Sex-based differences in ion channel subunit expression from non-diseased ventricles^1^**. Ratios are relative to the male endocardial cell.

Supplementary Table S2 **Effects of estradiol on *I*_Kr_**.

Supplementary Table S3 **Effects of testosterone on *I*_Ks_ and *I*_Ca,L_**.

Supplementary Table S4 **Effects of progesterone on *I*_Ks_ and *I*_Ca,L_**.

Supplementary Table S5 **Conduction velocity**.

Supplementary Table S6 **QT intervals comparison**.

Supplementary Figure S1 **Simulated action potentials (APs) for the 1000th paced beat at a cycle length of 1000 ms in single epicardial cells**. Action potential durations (APDs) with no hormone (HM) addition are calculated using the index value (also shown in Figure 1B – red bars) from Table S1 (see Methods). **(A)** Effects of two physiological concentrations of male hormone (DHT **–** 10 and 35 nM) in male cells. **(B)** Estrogen and progesterone at different physiological concentrations corresponding to three stages of the menstrual cycle were added in female cells: early follicular phase (estrogen: 0.1 nM and progesterone: 2.5 nM), late follicular phase (estrogen: 1 nM and progesterone: 2.5 nM) and luteal phase (estrogen: 0.7 nM and progesterone: 40.6 nM). The APD for each case is indicated.

Supplementary Figure S2 **Modeling genomic and acute hormone effects on electrical restitution in single cells**. **(A)** APD restitution curves generated with S1–S2 pacing protocol is shown. **(B)** Slope of APD restitutions. Gender, cell types, and concentrations of sex steroid hormone are indicated.

Supplementary Figure S3 **Calculated 5000 cases of conduction velocity using the index ratio of gap-junction for each model (see Methods)**. The ratio of ion channel conductance was randomly chosen within one standard deviation of experimental data (Table S1).

Supplementary Figure S4 **5000 cases of conduction velocity were simulated within a standard deviation of experimental data (Table S1) in male and female models**. Ion channel conductance was fixed at the index values. The results of using the index ratio of gap-junction are shown in red bars.

Supplementary Figure S5 **Two-dimensional transmural tissues from male model in the absence or presence of DHT or with drug addition show linear reductions in APDs from endocardium to epicardium**. APDs are indicated by color gradient.

Supplementary Figure S6 **Simulated transmural linear APD gradients in female model through the menstrual cycles (early follicular, late follicular, and luteal) and during late follicular phase with drug application**. APDs are indicated by color gradient.

Supplementary Figure S7 **Simulated reentry wave on female heterogeneous tissues (Figure S6) in the presence of female hormones (B–D) and *I*_Kr_ block during early follicular (A) and luteal phases (B)**. Seven snapshots following application of hormones and/or drug at indicated time points. The same protocol as in Figure 4 was used.

Supplementary Figure S8 **Simulated APs under SNS stimulations for the 1000th paced beat at a cycle length of 1000 ms in single endocardial and epicardial cell**. Estrogen and progesterone at different physiological concentrations corresponding to three stages of the menstrual cycle were added in female cells: early follicular phase (estrogen: 0.1 nM and progesterone: 2.5 nM), late follicular phase (estrogen: 1 nM and progesterone: 2.5 nM) and luteal phase (estrogen: 0.7 nM and progesterone: 40.6 nM). The APD for each case is indicated.
