# Supplementary material for: In silico Prediction of Sex-Based Differences in Human Susceptibility to Cardiac Ventricular Tachyarrhythmias
Source: Front Physiol. 2012 Sep 14;3:360. doi: 10.3389/fphys.2012.00360 (PMC3442371; doi:10.3389/fphys.2012.00360)
Supplement: Supplementary Table S1 — Sex-based differences in ion channel subunit expression from non-diseased ventricles1. Ratios are relative to the male endocardial cell. [file Data_Sheet_1.PDF]

SUPPLEMENTAL MATERIAL

Supplemental Detailed Methods

Table S1: Sex-based differences in ion channel subunit expression from non-diseased ventricles [1]. Ratios are relative to the male endocardial cell.

Channels in the model	Gene	epi		endo	
		Male	Female	Male	Female
I_{Ks}	**KvLQT1**	104.2	77.5	90.4	109.6
	**MinK (KCNE1)**	13.6±1.4 *↑	7.3±5 *↓	11.9±4.2 **↑	5.8±2.6 **↓
	Ratio	*1.04 ± 0.04*	*0.87 ± 0.14*	*1 ± 0.12*	*0.83 ± 0.07*
	**Functions**	Co-express KvLQT1 & MinK shifts the voltage to more positive voltages. Also increase amplitude of expressed current. Only MinK shows significant differences between male and female. The stoichiometry of KCNE1:KCNQ1 in I_{Ks} channels is a fixed 2:4 [2]. The ratio was calculated by modified the 1/3 of current activity according to MinK.			
I_{Kr}	**hERG (Kv11.1)**	179.5±6.4 *↑	144.2±41.1 *↓	164.8±54.9 ↑	130.5±65 ↓
	Ratio	*1.09 ± 0.039*	*0.875 ± 0.25*	*1 ± 0.33*	*0.79 ± 0.39*
I_{K1}	**Kir2.1**	94.5	93.8	104.1	79.7
	**Kir2.2**	111.1	115.3	93.6	104.5
	**Kir2.3**	91.2±31.8 *↑	21.4±10.6 f *↓	92.7±26.8 **↑	55.2 ± 21.5 f **↓
	Ratio	*0.98 ± 0.12*	*0.74 ± 0.04*	*1 ± 0.09*	*0.86 ± 0.077*

	**Functions**	Kir2.x channels mediate cardiac I_{K1} [3], however only Kir2.3 is significantly different. Kir2.3 changes the 1/3 of channel activity.			
**$I_{to,s}$**	**Kv1.4**	12.1±3.3<br>m *↑	5.4±3.8<br>f *↓	20.2±4.0<br>m **↑	12.9±5.2<br>f **↓
	Ratio	*0.6 ± 0.16*	*0.26 ± 0.19*	*1 ± 0.2*	*0.64 ± 0.26*
**I_{NaK}**	**ATPase α1**	207.7±67<br>*↓	513.4±134.6<br>*↑	269.0±70.3<br>**↓	622.5±287.7<br>**↑
	**α3**	1481±267<br>*↑	917.8±416.7<br>*↓	1547.6±299.5<br>**↑	1014.2±294.5<br>**↓
	Ratio	*0.94 ± 0.18*	*0.7 ± 0.3*	*1.0 ± 0.2*	*0.79 ± 0.26*
	**Functions**	α3 is about 2-fold more activity than α1 in LV [4]. The ratio was calculated depended on 1/3 of α1 and 2/3 of α3.			
**I_{pCa}**	**PMCA1**	31.7	46.4	44.1	48.1
	**PMCA4**	377.0±57.2<br>*↓	682.1±265.9<br>*↑	426.8±116.6	685.2±379.7
	Ratio	*0.88 ± 0.13*	*1.6 ± 0.6*	*1.± 0.27*	*1.6± 0.89*
	**Functions**	PMCA1 serves a critical housekeeping function that requiring for the maintenance of basic cellular function. [5]. PMCA4 nearly ubiquitous distribution has similar role as PMCA1. PMCA4 was reported much more activity than PMCA1 in LV [4], and there are no differences between genders in PMCA1. The ratio was calculated depended only on the PMCA4.			
**I_{up}**	**SERCA2**	4850.5±146<br>m *↓	6728.4±1876.1<br>f *↑	3410.4±982.1<br>m	3921.9±1760.7<br>f
	**Ratio**	*1.42 ± 0.04*	*1.97 ± 0.55*	*1± 0.28*	*1.15± 0.5*

**Calmodulin**	**CALM1**	1329.5	991.9	879.8	1122.7
	**CALM3**	1326.9±220<br>* ↓	1955.5±372.2<br>* ↑	1206.9±187.7<br>** ↓	1600.5±242.9<br>** ↑
	Ratio	*1.07 ± 0.12*	*1.41 ± 0.2*	*1± 0.1*	*1.21± 0.14*
	**Functions**	CALM3 is more activity than CALM1 (~2-fold in LV) [4]. The ratio was obtained by changing the 2/3 of activity depends on CALM3.			
**Gap-junction**	**Cx43**	1124.1±357<br>* ↑	728.6±274.1<br>* ↓	1196.3±311.7<br>** ↑	810.9±333.9<br>** ↓
	Ratio	*0.94 ± 0.3*	*0.61 ± 0.24*	*1.0 ± 0.26*	*0.68 ± 0.28*

Acute effects of sex-steroid hormones on ion channels

We simulated the I_{Kr} current with addition of estrogen and/or drug addition by scaling the conductance of current. Currents were scaled to the experimentally measured ratio of conductance.

Table S2: Effects of estradiol on I_{Kr}

	**Estradiol (E2)**			**Estradiol (E2) + E-4031 10 nM**		
**Channel**	0.1 nM	0.7 nM	1 nM	0.1 nM	0.7 nM	1 nM
$\mathbf{I_{Kr}}$	0.98	0.9	0.86	0.9	0.678	0.678
**REF**	[6]					

Progesterone and testosterone affect the conductance of I_{Ks} but have no distinguishable effects on its kinetics. When progesterone and testosterone were applied, we multiplied the conductance of I_{Ks} and $I_{Ca,L}$ by scaling factors as follows:

Table S3: Effects of Testosterone on I_{Ks} and $I_{Ca,L}$

	**Testosterone (DHT)**			**DHT + E-4031 10 nM**
**Channel**	10 nM	35 nM	**Channel**	35 nM
I_{Ks}	1.38	1.4	I_{Kr}	0.9
I_{CaL}	0.94	0.8		
**REF**	[7]		**REF**	[6]

Table S4: Effects of progesterone on I_{Ks} and $I_{Ca,L}$

	**Progesterone (baseline)**		**Progesterone (SNS stimulations)**	
**Channel**	2.5 nM	40.6 nM	2.5 nM	40.6 nM
I_{Ks}	1.19	1.4	1.6568	1.672
I_{CaL}	1.0	1.0	1.0416	0.9296
**Kinetics of I_{CaL}**	No effects		dss=1.0/(1.0+exp((-(v+2.44))/4.730)) fss=1.0/(1.0+exp((v+21.28)/3.596))	dss=1.0/(1.0+exp((-(v-0.46))/5.230)) fss=1.0/(1.0+exp((v+23.28)/3.096))
**REF**	[8]			

Transmural fiber simulations

We simulated a transmural fiber composed of 360 ventricular cells ($\Delta x = \Delta y = 100$ μm) connected by resistances to simulate gap junctions [9]. The fiber contains an endocardial region (cells 1 to 160) and epicardial region (cells 161 to 360), which shown a linear decreased in APDs [10, 11]. G_{Kr} was used the index value of endocardium in the cell #1, and the index value of epicarium in the cell #360. In the female model, G_{Kr} was monotonically increased from 0.036 to 0.042. In the male model, G_{Kr} was linearly increased from 0.046 to 0.05. AP simulations were carried out in epi-/endocardial cells by changing various ion channel conductance and gap-junctions (see Table S1). The fiber was paced at BCL = 1000 ms for 500 beats. Conduction velocities for each model were shown in Table S5.

Table S5: Conduction Velocity

	**ENDO**	**EPI**	**Transmural**
	**cm/s**		
**Male**	66	64	65
**Female**	53	50	51

ECG computation

Extracellular unipolar potentials (Φ_e) generated by the fiber in an extensive medium of conductivity σ_e, were computed from the transmembrane potential V_m using the integral expression as in Gima and Rudy [12]:

$$\Phi_e(x',y',z') = \frac{a^2\sigma_i}{4\sigma_e}\int(-\nabla V_m)\bullet\left[\nabla\frac{1}{r}\right]dx$$

$$r = [(x-x')^2 + (y-y')^2 + (z-z')^2]^{1/2}$$

where ∇V is the spatial gradient of V_m, a is the radius of the fiber, σ_i is the intracellular conductivity, σ_e is the extracellular conductivity, and r is the distance from a source point (x, y,

z) to a field point (x', y', z'). Φ_e was computed at an "electrode" site 2.0 cm away from the distal end along the fiber axis. QT intervals were shown in Table S6 for each cell type and transmural cables.

Table S6: QT intervals comparison

	**ENDO**	**EPI**	**Transmural**
Male	**ms**		
**No HM (NH)**	348	330	318
**DHT 10 nM**	345	328	315
**DHT 35 nM**	340	324	310
**DHT 35 nM + drug**	357	342	325
Female			
**No HM (NH)**	408	400	354
**Early follicular**	415	405	355
**Late follicular**	435	425	375
**Luteal**	420	412	365
**Late follicular + drug**	480	470	420

Alternans calculation

Single epicardial cells were constantly pacing at a basic cycle length between 180 and 330 ms for 1000 beats. AP duration (APD) was measured as the time between the time of the maximum AP upstroke velocity and 90% cellular repolarization.

In 1D cable simulations, transmural fibers and endo cable were constantly pacing at a fast rate for 200 beats. For the pacing down protocol, transmural fiber was paced at a BCL = 500 ms to 100 beats, and the BCL was then shortened by 10-ms until alternans appears, and then stepped down 1-ms intervals until conduction block.

Transmural tissue simulations

We simulated a heterogeneous cardiac tissue on 360 by 440 with $\Delta x = \Delta y = 150$ µm. The tissue contains an endocardial region (fibers 1 to 160) and epicardial region (fibers 161 to 360). Channel conductance and gap-junctions parameters are same as in the one-dimensional simulations. Current flow is described by the following equation:

$$\frac{\partial V(x,y,t)}{\partial t} = D_x \frac{\partial^2 V(x,y,t)}{\partial x^2} + D_y \frac{\partial^2 V(x,y,t)}{\partial y^2} - \frac{I_{ion} - I_{stim}}{C_m}$$

Where V is the membrane potential, x and y are distances in the longitudinal and transverse directions, respectively, D_x and D_y are diffusion coefficients in the x and y directions. I_{stim} is 180 mA/cm^2 for 1 ms. We also incorporated anisotropic effects by setting D_x and D_y such that the ratio of conduction velocity is 1:2 [13].

The tissue was first paced for 50 beats (S1) at BCL = 1000 ms on the entire length of one side of tissue. In ***male*** cases, a premature stimulus (S2) was then delivered at 370 ms after S1 in a 2.7 cm × 1.5 cm area on the top edge of the endocardial region. In ***female*** cells, S2 was applied at various time points for each case indicated in Figure 5 and Figure 6.

Figure Legends

Figure S1 – Simulated action potentials (APs) for the 1000th paced beat at a cycle length of 1000 ms in single epicardial cells. Action potential durations (APDs) with no hormone (HM) addition are calculated using the index value (also shown in Figure 1B – red bars) from Table S1 (see **methods**). A: Effects of two physiological concentrations of male hormone (DHT –10 and 35 nM) in male cells. B: Estrogen and progesterone at different physiological concentrations

corresponding to three stages of the menstrual cycle were added in female cells: early follicular phase (estrogen: 0.1 nM and progesterone: 2.5 nM), late follicular phase (estrogen: 1 nM and progesterone: 2.5 nM) and luteal phase (estrogen: 0.7 nM and progesterone: 40.6 nM). The APD for each case is indicated.

Figure S2 – Modeling genomic and acute hormone effects on electrical restitution in single cells. A: APD restitution curves generated with S1S2 pacing protocol is shown. B: Slope of APD restitutions. Gender, cell types and concentrations of sex-steroid hormone are indicated.

Figure S3 – Calculated 5000 cases of conduction velocity using the index ratio of gap-junction for each model (see **methods**). The ratio of ion channel conductance was randomly chosen within one standard deviation of experimental data (Table S1).

Figure S4 – 5000 cases of conduction velocity were simulated within a standard deviation of experimental data (Table S1) in male and female models. Ion channel conductance was fixed at the index values. The results of using the index ratio of gap-junction are shown in red bars.

Figure S5 – Two-dimensional transmural tissues from male model in the absence or presence of DHT or with drug addition show linear reductions in APDs from endocardium to epicardium. APDs are indicated by color gradient.

Figure S6 – Simulated transmural linear APD gradients in female model through the menstrual cycles (early follicular, late follicular and luteal) and during late follicular phase with drug application. APDs are indicated by color gradient.

Figure S7 – Simulated reentry wave on female heterogeneous tissues (Figure S6) in the presence of female hormones (B-D) and I_{Kr} block during early follicular (A) and luteal phases (B). Seven snapshots following application of hormones and/or drug at indicated time points. The same protocol as in Figure 4 was used.

Figure S8 – Simulated APs under SNS stimulations for the 1000th paced beat at a cycle length of 1000 ms in single endocardial and epicardial cell. Estrogen and progesterone at different physiological concentrations corresponding to three stages of the menstrual cycle were added in female cells: early follicular phase (estrogen: 0.1 nM and progesterone: 2.5 nM), late follicular phase (estrogen: 1 nM and progesterone: 2.5 nM) and luteal phase (estrogen: 0.7 nM and progesterone: 40.6 nM). The APD for each case is indicated.

REFERENCE

1. Gaborit N, Varro A, Le Bouter S, Szuts V, Escande D, Nattel S, Demolombe S. Gender-related differences in ion-channel and transporter subunit expression in non-diseased human hearts. *J Mol Cell Cardiol*. 2010
2. Nakajo K, Ulbrich MH, Kubo Y, Isacoff EY. Stoichiometry of the kcnq1 - kcne1 ion channel complex. *Proc Natl Acad Sci U S A*. 2010;107:18862-18867
3. Dhamoon AS, Pandit SV, Sarmast F, Parisian KR, Guha P, Li Y, Bagwe S, Taffet SM, Anumonwo JM. Unique kir2.X properties determine regional and species differences in the cardiac inward rectifier k+ current. *Circ Res*. 2004;94:1332-1339
4. Gaborit N, Le Bouter S, Szuts V, Varro A, Escande D, Nattel S, Demolombe S. Regional and tissue specific transcript signatures of ion channel genes in the non-diseased human heart. *J Physiol*. 2007;582:675-693

5. Brini M. Plasma membrane ca(2+)-atpase: From a housekeeping function to a versatile signaling role. *Pflugers Arch*. 2009;457:657-664
6. Kurokawa J, Tamagawa M, Harada N, Honda SI, Bai CX, Nakaya H, Furukawa T. Acute effects of estrogen on the guinea pig and human ikr channels and drug-induced prolongation of cardiac repolarization. *J Physiol*. 2008
7. Bai CX, Kurokawa J, Tamagawa M, Nakaya H, Furukawa T. Nontranscriptional regulation of cardiac repolarization currents by testosterone. *Circulation*. 2005;112:1701-1710
8. Nakamura H, Kurokawa J, Bai CX, Asada K, Xu J, Oren RV, Zhu ZI, Clancy CE, Isobe M, Furukawa T. Progesterone regulates cardiac repolarization through a nongenomic pathway: An in vitro patch-clamp and computational modeling study. *Circulation*. 2007;116:2913-2922
9. Faber GM, Rudy Y. Action potential and contractility changes in [na(+)](i) overloaded cardiac myocytes: A simulation study. *Biophys J*. 2000;78:2392-2404
10. Glukhov AV, Fedorov VV, Anderson ME, Mohler PJ, Efimov IR. Functional anatomy of the murine sinus node: High-resolution optical mapping of ankyrin-b heterozygous mice. *Am J Physiol Heart Circ Physiol*. 2010;299:H482-491
11. Lou Q, Fedorov VV, Glukhov AV, Moazami N, Fast VG, Efimov IR. Transmural heterogeneity and remodeling of ventricular excitation-contraction coupling in human heart failure. *Circulation*. 2011;123:1881-1890
12. Gima K, Rudy Y. Ionic current basis of electrocardiographic waveforms: A model study. *Circ Res*. 2002;90:889-896
13. Frank K, Kranias EG. Phospholamban and cardiac contractility. *Ann Med*. 2000;32:572-578

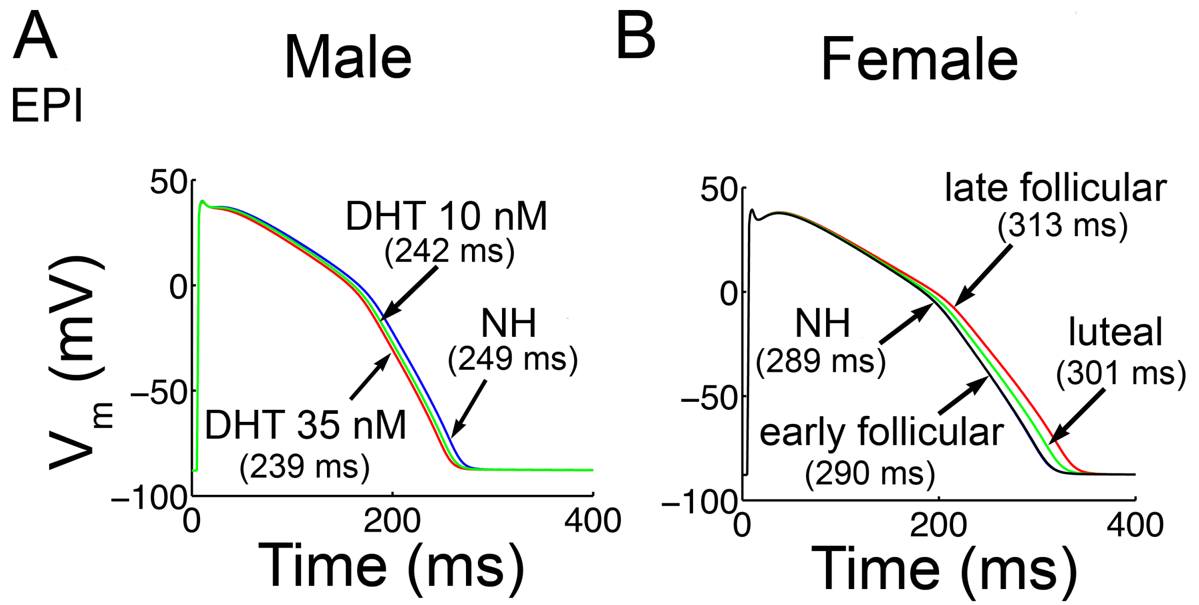
A
EPI
Male
B
Female
DHT 10 nM
(242 ms)
NH
(249 ms)
DHT 35 nM
(239 ms)
late follicular
(313 ms)
NH
(289 ms)
luteal
(301 ms)
early follicular
(290 ms)
Vm (mV)
Time (ms)
50
0
−50
−100
200
400

Figure S1

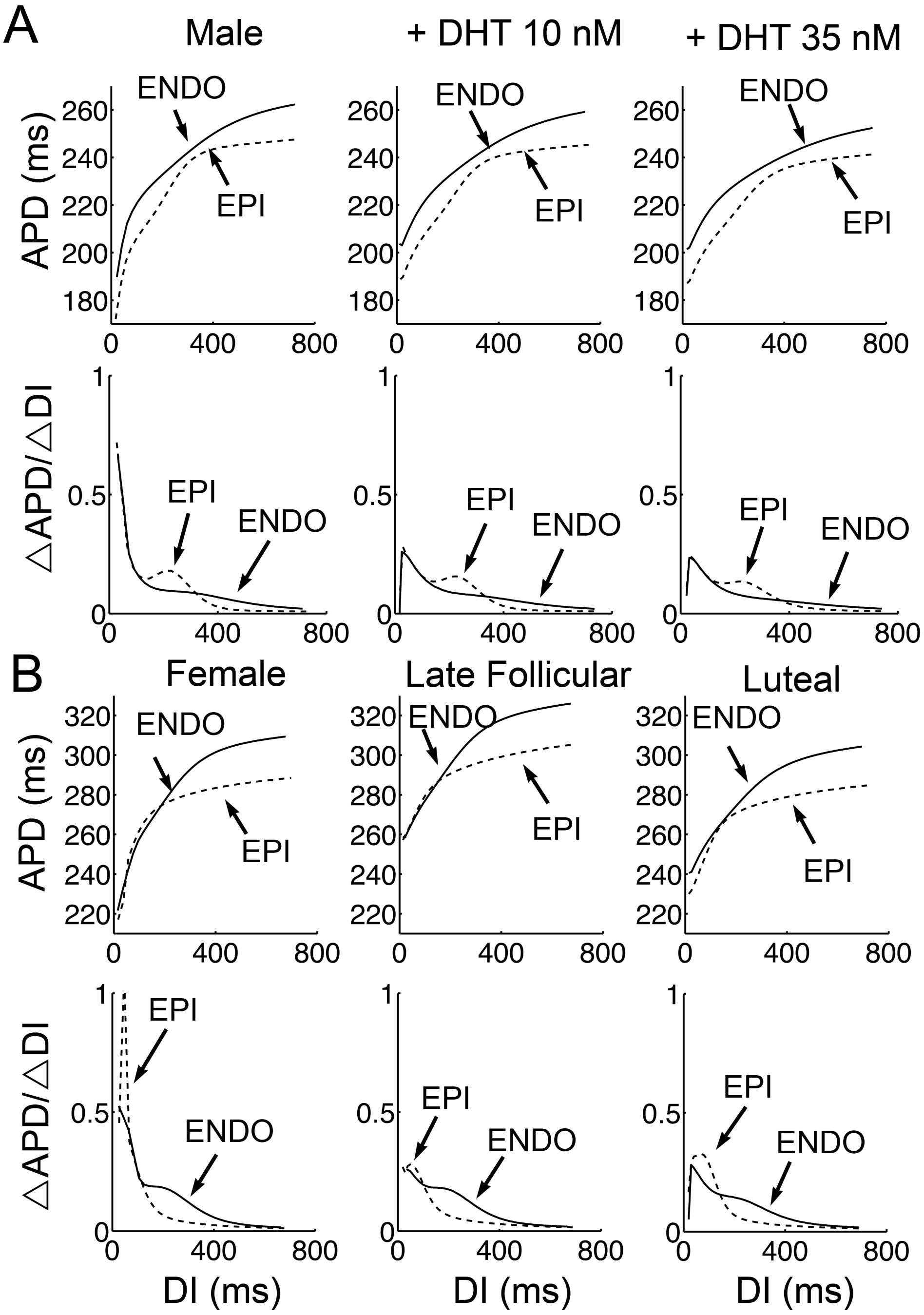
A
Male
+ DHT 10 nM
+ DHT 35 nM
ENDO
EPI
APD (ms)
△APD/△DI
B
Female
Late Follicular
Luteal
DI (ms)

Figure S2

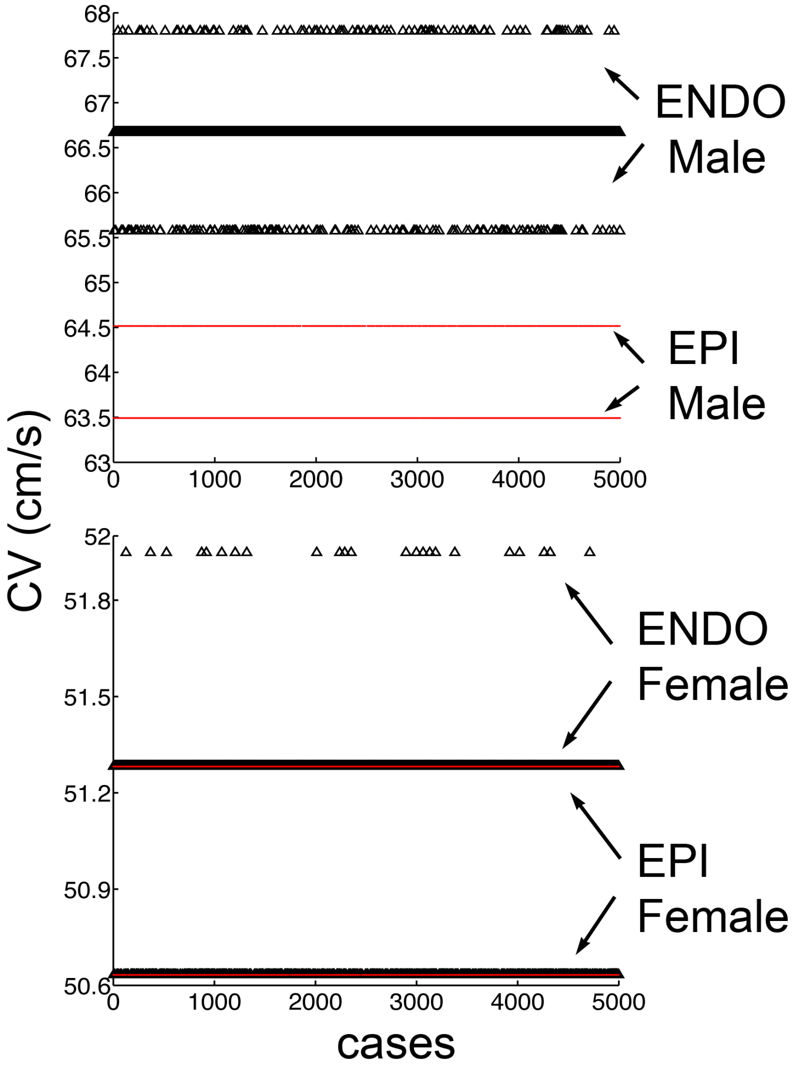
68
67.5
67
66.5
66
65.5
65
64.5
64
63.5
63
0
1000
2000
3000
4000
5000
ENDO
Male
EPI
Male
CV (cm/s)
52
51.8
51.5
51.2
50.9
50.6
ENDO
Female
EPI
Female
cases

Figure S3

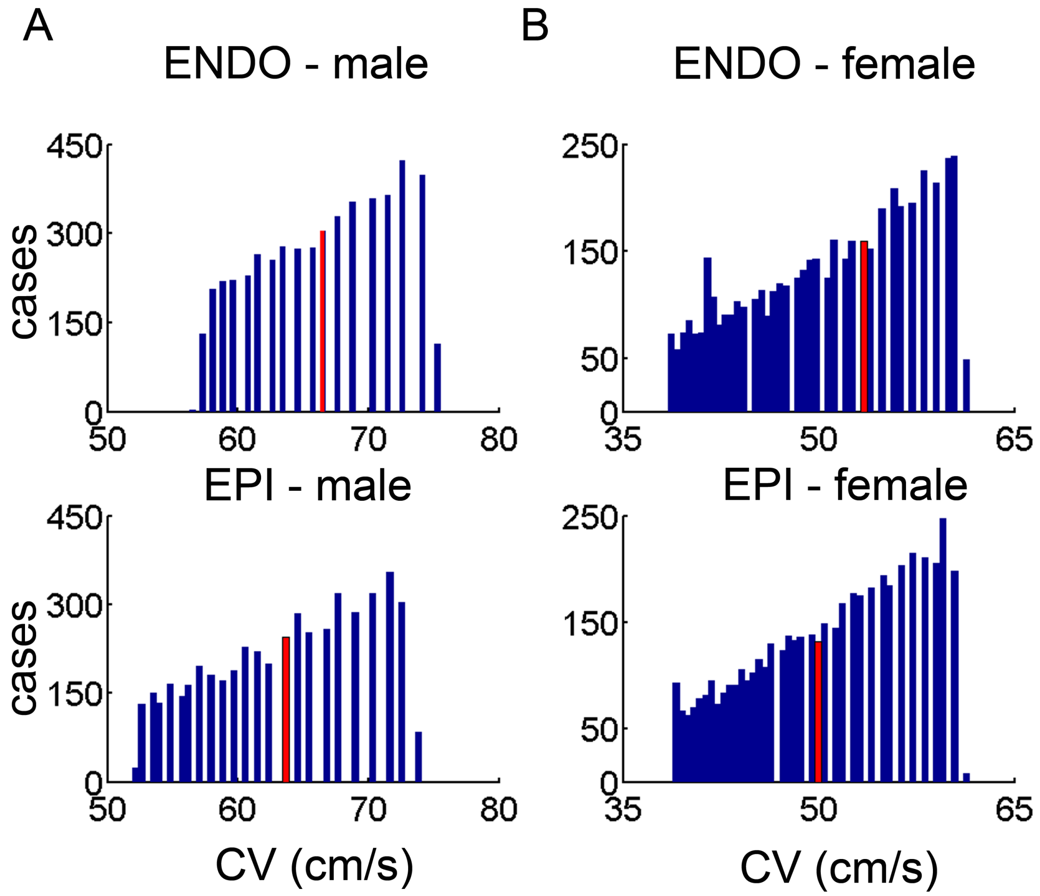
A
B
ENDO - male
ENDO - female
EPI - male
EPI - female
cases
cases
CV (cm/s)
CV (cm/s)
450
300
150
0
250
150
50
50
60
70
80
35
65

Figure S4

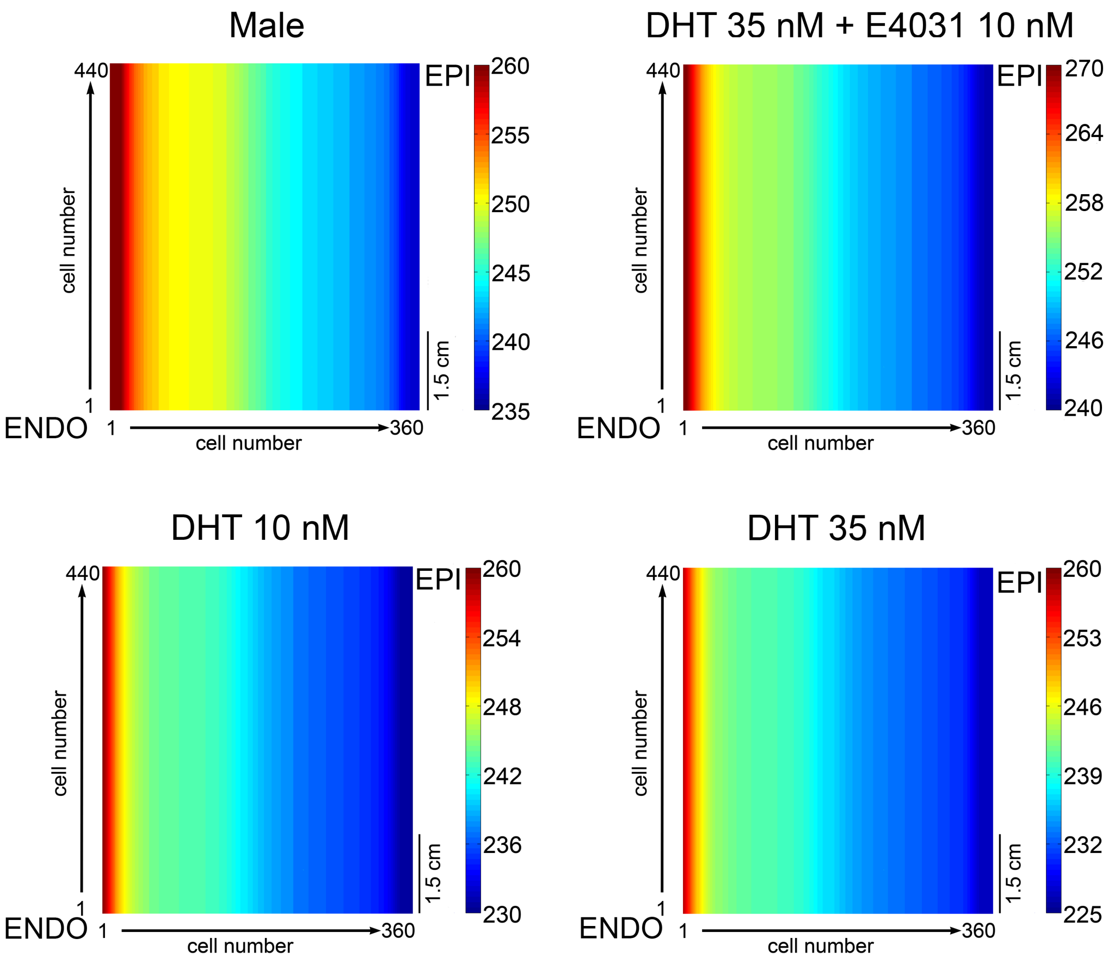

Male
440
EPI
260
255
250
245
240
235
cell number
1.5 cm
1
ENDO
1
cell number
360
DHT 35 nM + E4031 10 nM
440
EPI
270
264
258
252
246
240
cell number
1.5 cm
1
ENDO
1
cell number
360
DHT 10 nM
440
EPI
260
254
248
242
236
230
cell number
1.5 cm
1
ENDO
1
cell number
360
DHT 35 nM
440
EPI
260
253
246
239
232
225
cell number
1.5 cm
1
ENDO
1
cell number
360

Figure S5

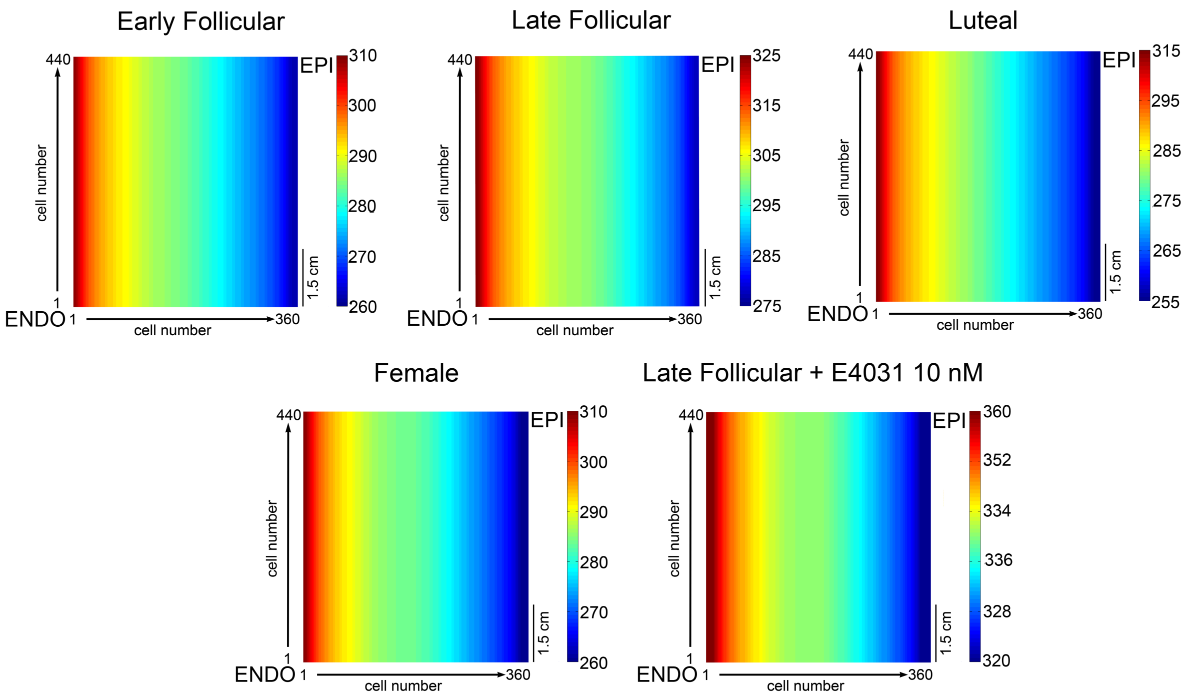
Early Follicular
440
cell number
1
ENDO
1
cell number
360
EPI
1.5 cm
310
300
290
280
270
260
Late Follicular
440
cell number
1
ENDO
1
cell number
360
EPI
1.5 cm
325
315
305
295
285
275
Luteal
440
cell number
1
ENDO
1
cell number
360
EPI
1.5 cm
315
295
285
275
265
255
Female
440
cell number
1
ENDO
1
cell number
360
EPI
1.5 cm
310
300
290
280
270
260
Late Follicular + E4031 10 nM
440
cell number
1
ENDO
1
cell number
360
EPI
1.5 cm
360
352
334
336
328
320

Figure S6

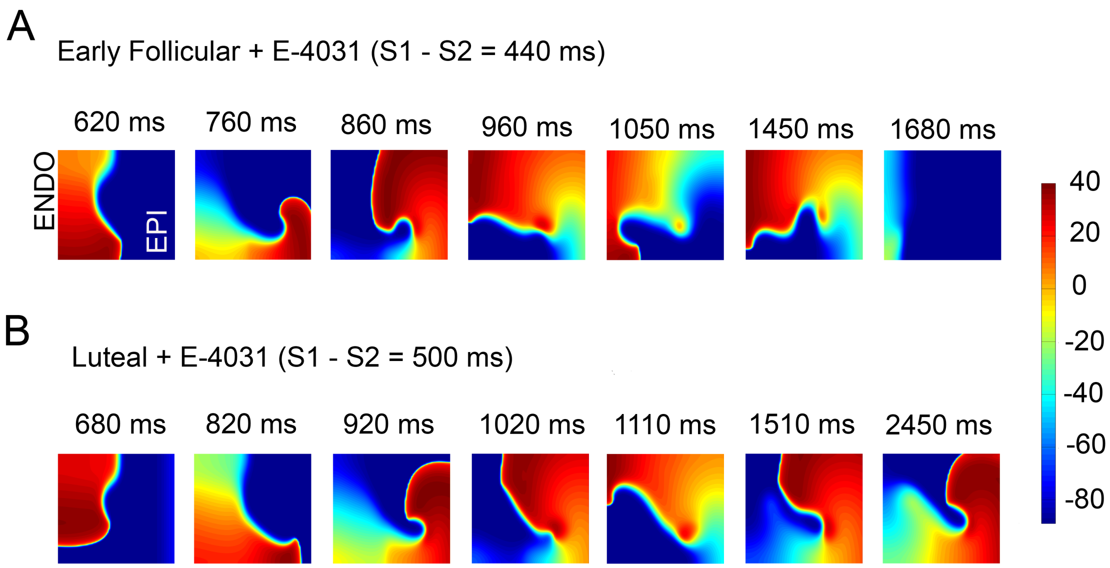
A
Early Follicular + E-4031 (S1 - S2 = 440 ms)
620 ms
760 ms
860 ms
960 ms
1050 ms
1450 ms
1680 ms
ENDO
EPI
B
Luteal + E-4031 (S1 - S2 = 500 ms)
680 ms
820 ms
920 ms
1020 ms
1110 ms
1510 ms
2450 ms
40
20
0
-20
-40
-60
-80

Figure S7

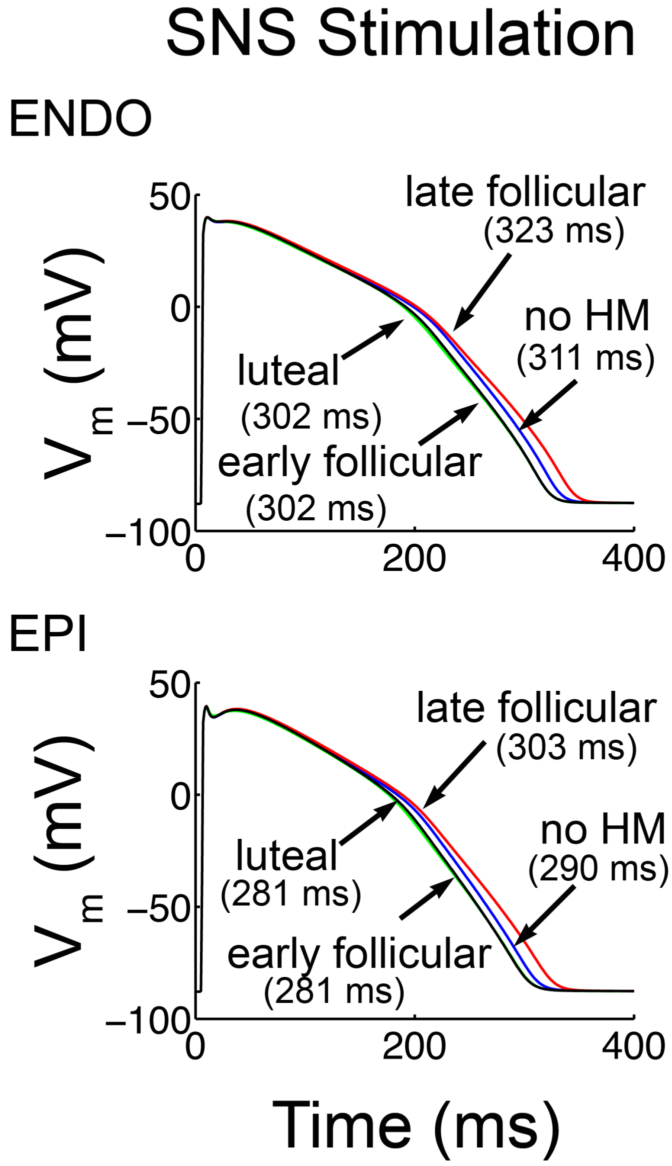
SNS Stimulation
ENDO
late follicular
(323 ms)
no HM
(311 ms)
luteal
(302 ms)
early follicular
(302 ms)
EPI
late follicular
(303 ms)
no HM
(290 ms)
luteal
(281 ms)
early follicular
(281 ms)
Vm (mV)
50
0
−50
−100
0
200
400
Time (ms)

Figure S8
